# What Are the Functions of Chitin Deacetylases in *Aspergillus fumigatus*?

**DOI:** 10.3389/fcimb.2020.00028

**Published:** 2020-02-06

**Authors:** Isabelle Mouyna, Sarah Dellière, Anne Beauvais, Fabrice Gravelat, Brendan Snarr, Mélanie Lehoux, Caitlin Zacharias, Yan Sun, Steven de Jesus Carrion, Eric Pearlman, Donald C. Sheppard, Jean-Paul Latgé

**Affiliations:** ^1^Aspergillus Unit, Pasteur Institut, Paris, France; ^2^Infectious Diseases in Global Health Program, Centre for Translational Biology, McGill University Health Centre, Montréal, QC, Canada; ^3^Departments of Microbiology and Immunology, Medicine, McGill University, Montréal, QC, Canada; ^4^Department of Ophthalmology and Visual Sciences, Case Western Reserve University, Cleveland, OH, United States; ^5^Department of Ophthalmology, University of California, Irvine, Irvine, CA, United States

**Keywords:** *Aspergillus fumigatus*, filamentous fungi, cell wall, chitin deacetylase, chitosan, chitin, conidia

## Abstract

Deacetylation of chitin by chitin deacetylases (Cda) results in the formation of chitosan. Chitosan, a polymer of β1,4 linked glucosamine, plays multiple roles in the function of the fungal cell wall, including virulence and evasion of host immune responses. In this study, the roles of chitosan and putative *CDA*s in cell wall structure and virulence of *Aspergillus fumigatus* were investigated. Low levels of chitosan were found in the conidial and cell wall of *A. fumigatus*. Seven putative *CDA* genes were identified, disrupted and the phenotype of the single mutants and the septuple mutants were investigated. No alterations in fungal cell wall chitosan levels, changes in fungal growth or alterations in virulence were detected in the single or septuple Δ*cda1-7* mutant strains. Collectively, these results suggest that chitosan is a minority component of the *A. fumigatus* cell wall, and that the seven candidate Cda proteins do not play major roles in fungal cell wall synthesis or virulence. However, Cda2 is involved in conidiation, suggesting that this enzyme may play a role in N-acetyl-glucosamine metabolism.

## Introduction

The fungal cell wall is a complex exoskeleton that protects the cell from osmotic pressure changes and other environmental stresses, yet allows the cell to interact with its environment. Importantly, the fungal cell wall constitutes the first interface between pathogenic fungi and their host. Its components are fungal-specific and are therefore ideal targets for recognition as non-self by immune cells as well as ideal targets for antifungals drugs (Lee and Sheppard, 2016; van de Veerdonk et al., 2017).

Chitin, a homopolymer of N-acetyl-glucosamine (GlcNAc) linked in ß1,4, is produced by many living organisms including crustaceous, insects, and fungi. Chitin can comprise up to 20% of the inner fungal cell wall and can be partially de-N-acetylated to form chitosan, a polymer containing glucosamine (GlcN) as well as GlcNAc. The distinction between these two molecules is not absolute. Polymers with 50% or more acetylation are generally termed chitin, while those with acetylation levels less than this are commonly referred to as chitosan (Kasaai, 2009). Chitin deacetylation is catalyzed by a family of conserved carbohydrate esterase enzymes of type 4 (CE-4 family) known as chitin-deacetylases (E.C. 3.5.1.41) (Pochanavanich and Suntornsuk, 2002). Deacetylation alters the physical properties of the polymer to enhance solubility, flexibility, and confers a positive charge at neutral pH (Wang et al., 2016). A dynamic equilibrium between the quantity of chitin and chitosan exist in the cell wall but the environmental factors and regulatory elements governing this equilibrium are largely unknown.

The role of chitin deacetylation was first described in non-pathogenic yeast. In *Saccharomyces cerevisiae* (Christodoulidou et al., 1996) two functionally redundant *CDAs* were found to deacetylate chitin specifically in the ascospore cell wall, as the double Δ*cda1cda2* mutant exhibited a complete loss of chitosan from the ascospore. In *Schizosaccharomyces pombe*, a single chitin deacetylase, Cda1, is required for proper spore formation (Matsuo et al., 2005).

The role of chitosan in pathogenicity has been best studied in the fungal plant pathogens *Puccinia graminis, Colletotrichum graminicola* (El Gueddari et al., 2002), and more recently in *Magnaporte oryzae* (Geoghegan and Gurr, 2016, 2017; Kuroki et al., 2017). During infection with these organisms, chitin is deacetylated to chitosan within the appressorium, a flattened thickened hyphal tip by which these fungi attach to, and penetrate their host (El Gueddari et al., 2002). Conversion of chitin into chitosan is hypothesized to protect the appressoria from hydrolytic attack by chitinases present in the plant tissue. This approach also serves to prevent the detection of chitin from plant pattern recognition receptors such as CEBiP (Chitin Elicitor Binding Protein) to evade plant immunity (Geoghegan and Gurr, 2016). *M. oryzae* mutants lacking *CDAs* are unable to produce appressorium *in vitro*. However, appressorium development was restored in the mutant during infection of rice leaves and the mutant retained the ability to cause infection, suggesting other polymers may play a compensatory role *in planta* (Geoghegan and Gurr, 2016). Six *CDAs* were found in the genome of *A. nidulans* (Liu et al., 2017). However, in the absence of mutants, the role of *A. nidulans CDAs* could not be determined.

The role of chitosan in human pathogenic fungi has been well-studied in the yeast *Cryptococcus neoformans* which causes meningoencephalitis in immunocompromised patients. Chitosan is an important component of the *C. neoformans* vegetative cell wall (Baker et al., 2007). Three genes, *CDA1, CDA2*, and *CDA3*, were identified as coding for putative secreted Cdap in this yeast. Mutants lacking these genes were devoid of cell wall chitosan and exhibited increased susceptibility to cell wall stressors suggesting a role for chitosan in fungal cell wall integrity (Baker et al., 2007). Additionally, the triple Δ*cda* mutant (Δ*cda123*) exhibited attenuated virulence in a mice model of infection (Baker et al., 2011). It has been also reported recently that *CDA1* was required for fungal virulence (Upadhya et al., 2018). Accordingly, Upadhya et al. (2016) showed that protective immunity was induced in mice vaccinated with heat-killed Δ*cda123* cells and was effective in multiple mouse strains. The role of chitosan in other human fungal pathogens such as *A. fumigatus* has not been studied, although two putative chitin deacetylase genes have been annotated within the fungal genome, Afu4g09940 (*CDA5*) and Afu6g10430 (*CDA6*) (Gastebois et al., 2009).

Based on early studies in filamentous phytopathogenic ascomycetes, we hypothesized that chitosan might play a role in cell wall morphogenesis and in host-pathogen interactions in *A. fumigatus*. We explored this hypothesis with the following three specific questions: (i) is chitosan present in the mycelial or conidial cell wall of *A. fumigatus*? (ii) how many *CDAs* genes are present in *A. fumigatus* genome and (iii) what is the role of these *CDA*s in *A. fumigatus*? To answer these questions, we characterized the percentage of deacetylation of the chitosan in the mycelium and conidia. We constructed mutants deficient in *CDA*s and studied the effects of these gene deletions on mycelial and conidial cell wall integrity, morphogenesis, adherence, biofilm formation, and virulence.

## Materials and Methods

### Fungal Strains and Growth Media

The fungal parental strains used in this study was akuBKu80 pyrG+ (KU80) deficient for non-homologous end joining recombination (da Silva Ferreira et al., 2006) that was derived from the *A. fumigatus* strain CEA10 and retained the same virulence potential. Transformations were performed on minimal medium (Glc-MM) (10 g/L glucose (Glc), 0.92 g/L ammonium tartrate, 0.52 g/L KCl, 0.52 g/L MgSO4·7H_2_O, 1.52 g/L KH_2_PO_4_, 1 mL/L trace element solution (Cove, 1966), pH adjusted to 7.0). Hygromycin B (hph) (Sigma®, St Louis, MO, USA Sigma, Kawasaki, Japan) was added to transformation plates in an overlay after one night of incubation at room temperature, resulting in a final concentration of 150 μg/mL. For DNA extraction, cultures were grown in Sabouraud liquid medium (2% glucose + 1% mycopeptone) and DNA was isolated from *A. fumigatus* as previously described (Girardin et al., 1993). Strains constructed for this work are listed in the Table 1. Fungal strains were grown on different culture media: 2% malt-agar, Yeast Potato Dextrose (YPD) (1% yeast extract, 2% peptone, 2% dextrose), Glc-MM, GlcNAc-MM (MM in which Glc was replaced by GlcNAc), Brian (Brian et al., 1961), RPMI (Invitrogen) buffered with 34.53 g/L of MOPS or Dulbecco's Modified Eagle Medium (DMEM) (Invitrogen). Solid media was obtained by supplementation with 1.5–2% agar. Conidia were collected from agar slants/plates after 7 days of growth at room temperature using 0.05% Tween 20 solution.

**Table 1:** Fungal strains used and constructed in the present study.

**Strains**	**Genotypes**	**Source**
Parental strain KU80	CEA17Δ*akuB*^KU80^	da Silva Ferreira et al., 2006
Δ*cda1*	CEA17_Δ*akuB*^KU80^Δ*cda1::six-β-rec-hygroR-six*	This study
Δ*cda2*	CEA17_Δ*akuB*^KU80^Δ*cda2::six-β-rec-hygroR-six*	This study
Δ*cda3*	CEA17_ΔakuB ^KU80^Δcda3::six-β-rec-hygroR-six	This study
Δ*cda4*	CEA17_Δ*akuB*^KU80^Δ*cda4::six-β-rec-hygroR-six*	This study
Δ*cda5*	CEA17_Δ*akuB*^KU80^Δ*cda5::six-β-rec-hygroR-six*	This study
Δ*cda6*	CEA17_Δ*akuB*^KU80^Δ*cda6::six-β-rec-hygroR-six*	This study
Δ*cda7*	CEA17_Δ*akuB*^KU80^Δ*cda7::six-β-rec-hygroR-six*	This study
Δ*cda4/5*	CEA17_Δ*akuB*^KU80^ Δ*cda4::six/*Δ*cda5::six-β-rec-hygroR-six*	This study
Δ*cda4/5/6*	CEA17_Δ*akuB*^KU80^ Δ*cda4::six/*Δ*cda5::six/*Δ*cda6::six-β-rec-hygroR-six*	This study
Δ*cda4/5/6/3*	CEA17_Δ*akuB*^KU80^Δ*cda4::six/*Δ*cda5::six/*Δ*cda6::six/*Δ*cda3::six-β-rec-hygroR-six*	This study
Δ*cda4/5/6/3/2*	CEA17_Δ*akuB*^KU80^Δ*cda4::six/*Δ*cda5::six/*Δ*cda6::six/*Δ*cda3::six/*Δ*cda2::six-β-rec-hygroR-six*	This study
Δ*cda4/5/6/3/2/1*	CEA17_Δ*akuB*^KU80^Δ*cda4::six/*Δ*cda5::six/*Δ*cda6::six/*Δ*cda3::six/*Δ*cda2::six*Δ*cda1::six-β-rec-hygroR-six*	This study
Δ*cda4/5/6/3/2/1/7*(Δ*cda1-7*)	CEA17_Δ*akuB*^KU80^Δ*cda4::six/*Δ*cda5::six/*Δ*cda6::six/*Δ*cda3::six/*Δ*cda2::six*Δ*cda1::six*Δ*cda7::six-β-rec-hygroR-six*	This study

### Chitin Deacetylation Analysis

Two methods were used to quantify the degree of chitin deacetylation in *A. fumigatus* mycelium and conidia. First, the level of deacetylated chitin was measured in mycelial and conidial alkali-insoluble-(AI) and soluble-(AS) cell wall fractions by gas chromatography. Briefly, conidia were recovered with 0.05% tween 20 from 3 weeks old malt tubes at room temperature, and filtered using BD Falcon filters (BD Biosciences) to remove mycelium. Mycelium was obtained after growing conidia for 6 h at 37°C in Sabouraud medium to obtain germ tubes, which were inoculated in shaken flasks in RPMI-MOPS medium for 16 h at 37°C. Mycelium and conidia were disrupted with 1 and 0.17 mm diameter glass beads, respectively, for 2 min in Fast-prep cell breaker (MP Biomedical) at 4°C. The cell wall was recovered by centrifugation, washed three times in distilled water and lyophilized. Freeze-dried cell walls were boiled 10 min, twice in 50 mM Tris-HCl pH 7.4 containing 50 mM EDTA, 2% SDS and 40 mM β-mercaptoethanol, and extensively washed with water. The AS and AI fractions were extracted as described previously (Beauvais et al., 2005). For chitin deacetylation measurement, 5 mg AS and AI were incubated in 1 mL 2 M NaNO_2_ and 0.33 mL 2 N HCl for 6 h at room temperature. After evaporation of HNO_2_ under nitrogen for 30 min and centrifugation, the supernatant was loaded on 50 × 8 (H^+^) and 1 × 8 resin (acetate-O^−^), separated by a GF/C whatman filter. The water-eluted fraction was recovered and lyophilized. Half of the eluted fraction was reduced and peracetylated prior anhydromannitol quantification (representing the deacetylated chitin fraction degraded by HNO_2_) by gas chromatography (GC) (Sawardeker and Sloneker, 1965). The remaining half of the eluted fraction was hydrolyzed by 8 N HCl for 4 h at 100°C, reduced and peractylated prior determination of the total GlcN (by gas chromatography with meso-inositol as internal standard (Mouyna et al., 2010). The results were expressed in μg of deacetylated chitin in AI and AS fractions.

The degree of chitin deacetylation was also quantified using a colorimetric approach based on the specific absorbance of the free amine groups present on GalN sugars (Dubois, 1956). Briefly, 200 μL (10 mg/mL) cell wall were incubated in 200 μL 5% KHSO_4_ + 200 μL 5% NaNO_2_ for 1 h at 50°C. As a control, the cell wall was incubated in 5% NaCl instead of 5% NaNO_2_. After evaporation of HNO_2_ using 200 μL 12.5% ammonium sulfamate for 5 min, 200 μL 0.5% 3-methyl-2-benzothiazolinone hydrazine hydrochloride hydrate was added to the mixture. After incubation 30 min at 37°C, 200 μL 0.5% FeCl_3_ was added and the incubation was continued for 5 min. The OD of the extract was measured at 650 nm (800 nm as reference), using GlcN for the calibration curve. In parallel, 200 μL (10 mg/mL) conidial cell wall were hydrolyzed by 8 N HCl as above. The extract was then dried, solubilized in 200 μL of water and the amount of GlcN was quantified by HPLC with a CarboPAC PA-1 column (Dionex) and a pulsed electrochemical detector as previously described (Hartland et al., 1996).

### Construction of the Δ*cda* Deletion Strains

The single and multiple *CDA* deletion mutants were constructed in KU80 background (da Silva Ferreira et al., 2006) using the β-rec/six site-specific recombination system (Hartmann et al., 2010). The self-excising β-rec/six blaster cassette containing the hph resistance marker was released from the plasmid pSK529 via *Fsp*I restriction enzyme. Using the GeneArt® Seamless Cloning and Assembly (Life technologies, Carlsbad, CA 92008 USA) the *CDA* replacement cassette containing the marker module flanked by 5′ and 3′ homologous regions of the target gene generated by PCR (Table S1), was cloned into the pUC19 vector. The corresponding replacement cassettes were released from the resulting vector via *Sma*I or *Fsp*I. The KU80 parental strain was transformed with the *CDA* replacement cassette by electroporation as described by Sanchez and Aguirre to generate the single deletion mutants (Sánchez and Aguirre, 1996). For the construction of multiple deletion strains, single deletion strain was cultivated in presence of 2% xylose-containing MM (instead of glucose) that allows the excision of the selection marker by recombination of the six recognition regions. All gene deletions were confirmed by Southern blotting (Figure S1).

### Growth, Sporulation, Germination, and Morphology of the Δ*cda* Mutant Strains

Fungal growth was measured at 37or 50°C in agar based media: malt, Sabouraud, RPMI, Glc-MM, and GlcNAc-MM. Plates were spotted at the center with 10^5^ conidia suspended in 5 μL of 0.01% Tween 20 and the diameter of the colony was measured daily. Growth of the parental and mutant strains were also monitored at 37°C in 50 mL (3% glucose + 1% yeast extract) liquid medium shaken at 150 rpm and the dry weight determined.

Conidiation rates were quantified by inoculating 10^5^ conidia in tubes containing 2% Malt-agar medium. After 1 week of growth at RT, conidia were recovered with 5 mL of an aqueous 0.05% Tween 20 solution and the absorbance was measured at 600 nm (OD_600_ nm = 0.620 ≈ 2 × 10^7^ conidia/mL). Conidial germination was followed microscopically every 30 min after 4 h of growth at 37°C (inoculation of 5 μL of conidia at 10^6^ cell/mL on a glass slide with 2% Malt-agar medium). For the viability assays, conidia on agar slants were stored at 37°C for 2 months, conidial suspensions were then recovered in 0.05% Tween 20 water, and the viability of conidia was evaluated by quantitative culture on 2% malt medium.

### Susceptibility to Antifungal Drugs

The susceptibility of mutant strains to antifungal cell wall drugs was determined by spotting 3 μL of a suspension of conidia at 5 × 10^6^ cell/mL on plates supplemented with each drug. The sensitivity to Congo Red® (CR) and CFW was estimated on Glc-MM-agar containing 0–300 μg/mL CR or 0–200 μg/mL CFW. Plates were incubated for 48 h at 37°C and growth was measured. Resistance to caspofungin (0.07–38 μg/ml) was assessed in liquid RPMI-MOPS medium by the resazurin method (Clavaud et al., 2012).

### Biofilm Quantification

Biofilm cultures were grown statically for 20 h in tissue culture-treated 96 wells-plates (Falcon) in Brian media at 10^5^ conidia/mL as described previously (Snarr et al., 2017). Following incubation, non-adherent cells were removed by washing the wells twice with distilled water. The wells were stained with 0.1% (w/v) crystal violet for 10 min at room temperature and then rinsed twice with water. The remaining dye was extracted by addition of 70% (v/v) ethanol and left for 10 min, after which the absorbance was measured at 600 nm using Infinite M200 Pro spectrophotometer (Tecan).

### RNA Isolation and Gene Expression Level by Quantitative RT-PCR

The expression level of the sporulation involved-genes *BRLA, ABAA, WETA* was performed by reverse-transcriptase real-time PCR using primers designed using the Beacon Designer 4.0 software (Table S1). One hundred conidia were spotted on Glc-MM and GlcNAc-MM and let them grow 7 days at 37°C. Conidia from sporulating colonies were recovered in 500 μL phenol, disrupted with 0.5 mm diameter glass beads and RNA was isolated by using the phenol-chloroform method. Reverse transcription and real-time PCR were performed as described previously (Mouyna et al., 2010). The expression ratios were normalized to *TEF1* expression and calculated according to the ΔΔCt method (Livak and Schmittgen, 2001). Three independent biological replicates were performed.

### Virulence Studies

#### Murine Model of Invasive Pulmonary Aspergillosis

The virulence of the parental KU80 strain and the Δ*cda1-7* mutant was tested in a murine model of invasive pulmonary aspergillosis. Mice were immunosuppressed with cortisone acetate, 250 mg/kg subcutaneously on days−2 and +3, and cyclophosphamide (Western Medical Supply), 250 mg/kg intra-peritonealy on day−2 and 200 mg/kg on day +3 (Sheppard et al., 2004, 2006). For each fungal strain tested, groups of 16 mice were infected using an aerosol chamber as previously described (Sheppard et al., 2004). An additional 8 mice were immunosuppressed but not infected. To prevent bacterial infections, enrofloxacin was added to the drinking water while the mice were immunosuppressed. Mice were monitored for signs of illness and moribund animals were euthanized. All procedures involving mice were approved by the Animal Care Committees of the McGill University Health Center and followed the National Institutes of Health guidelines for animal housing and care. For fungal burden determination, 8 mice out of 16 were sacrificed 4 days after infection. The lungs were immediately homogenized in ice cold PBS containing 10 mL of protease inhibitor mix/mL (Sigma Aldrich), and then aliquoted and stored at −80°C until use. Pulmonary galactomannan (GM) content of lung homogenates was used as a surrogate measure of fungal burden using the Platelia Aspergillus EIA kit (Bio-Rad) as described previously (Gravelat et al., 2013). Lung homogenates were clarified by centrifugation, diluted 1:20 and assayed as per manufacturer's recommendations. Samples were compared to a standard curve composed of serial dilutions of lung homogenate from a mouse heavily infected.

#### Murine Model of *A. fumigatus* Keratitis

C57BL/6 mice were purchased from The Jackson Laboratory (Bar Harbor, ME) and AMCase^−/−^ mice were obtained from Dr. Lori Fitz through a material transfer agreement between UC Irvine and Pfizer. Mice were bred under specific pathogen-free conditions and maintained according to institutional guidelines. All animals were treated in accordance with the guidelines provided in the Association for Research in Vision and Ophthalmology (ARVO) statement for the Use of Animals in Ophthalmic and Vision Research, and were approved by Case Western Reserve University and UC Irvine IACUC committees.

Mice were infected as previously described (de Jesus Carrion et al., 2013; Leal et al., 2013). *A. fumigatus* strains were cultured in VMM agar in 25 cm^2^ tissue culture flasks. Dormant conidia were recovered with a bacterial L-loop and harvested in 5 ml PBS. Pure conidial suspensions were obtained by passing the culture suspension through PBS-soaked sterile gauges placed at the tip of a 10 ml syringe. Conidia were quantified and a stock was made at a final concentration of 2.5 × 10^4^ conidia/μl in PBS. Mice were anesthetized and the corneal epithelium was abraded with a 30 gauge needle to allow insertion of a 33-gauge Hamilton needle through which 2 μl PBS containing 4 × 10^4^ conidia were injected into the corneal stroma as described by (de Jesus Carrion et al., 2019). For assessment of fungal viability, whole eyes were homogenized under sterile conditions in 1 ml PBS, using the Mixer Mill MM300 (Retsch, Qiagen, Valencia, CA) at 33 Hz for 4 min. Subsequently, serial log dilutions were performed and plated onto Sabouraud dextrose agar plates (Becton Dickenson). Following incubation for 24 h at 37°C, the number of CFUs was determined by direct counting.

### Statistical Analyses and Graphs

All analyses were performed with GraphPad Prism software v6.0 (GraphPad Software). *P*-values were determined by student's *t*-test. A *P-*value of < 0.05 (two-tailed) is considered statistically significant.

## Results

### Quantification of Chitosan in the Cell Wall of *A. fumigatus*

**Figure 1:**
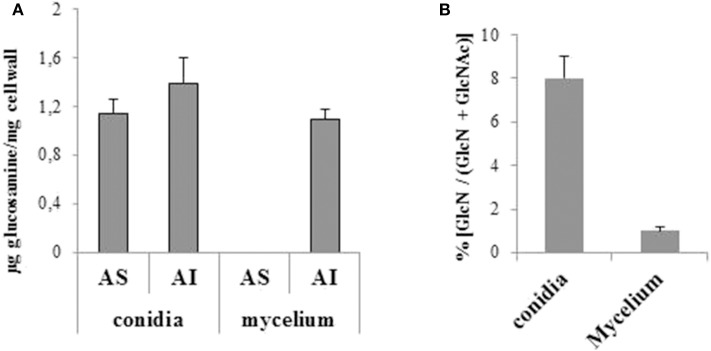
Quantification of glucosamine and N-acetyl glucosamine in the alkali-soluble and -insoluble fractions of mycelial and conidial cell wall. **(A)** Total amount of glucosamine determined by anhydromannitol quantification after HNO_2_ deamination followed by GC. **(B)** Percentage of deacetylation of chitin in the cell wall.

**Figure 2:**
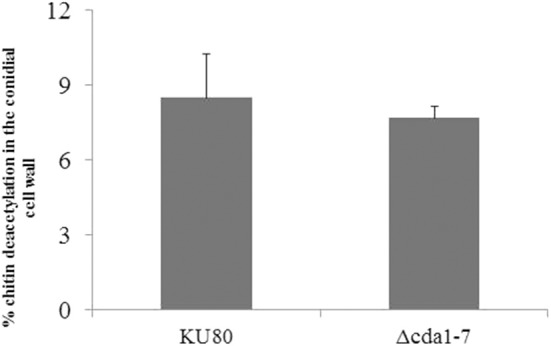
Percentage of chitin deacetylation in KU80 and Δ*cda1-7* conidial cell wall by the method of Dubois (1956).

Gas chromatography analysis of cell wall fractions from *A. fumigatus* revealed a total concentration of 1.1 μg/mg of free GlcN group in the mycelial cell wall, and 2.6 μg/mg in the the conidial cell wall (Figure 1A). These data indicate a chitin deacetylation fraction of 1.5% in the mycelium (AI fraction) and 7.8% in conidia (Figure 1B). Free amine quantification confirmed that 8.5% of conidial chitin is deacetylated (Figure 2). Unfortunately, due to the insufficient sensitivity of the method used to quantify free amine and to the low amount of deacetylated chitin in the mycelium, it was impossible to obtain reliable quantification of free amine on hyphal samples. Interestingly, glucosamine polymer was found in the AI and AS fractions of the conidial cell wall whereas it was only found in the AI fraction of the mycelial cell wall. Collectively these data suggest that chitosan is only a significant component of the conidial cell wall.

### Characterization of the *CDA* Gene Family in *A. fumigatus*

**Figure 3:**
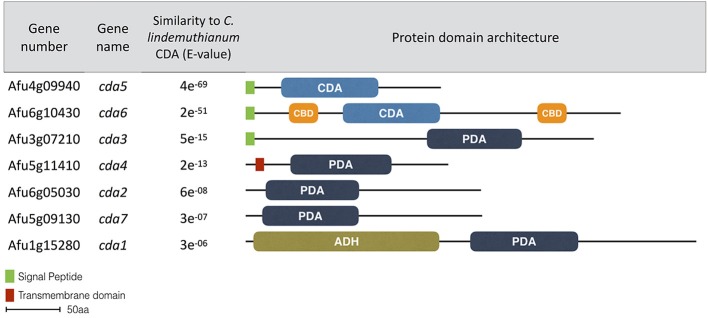
Bioinformatic analysis of *CDA* genes in fungi. Conserved domains and architecture of seven putative chitin de-N-acetylase in *A. fumigatus*. CDA, chitin deacetylase domain; PDA, polysaccharide deacetylase domain; CBD, chitin binding domain; ADH, deshydrogenase domain (Pfam 00106).

To identify putative *CDA-*encoding genes in *A. fumigatus*, the sequence of the well-characterized fungal chitin deacetylase from *Colletotrichum lindemuthianum* (AY633657) (Blair et al., 2006), was used to perform a BLAST search of the *A. fumigatus* genome (https://blast.ncbi.nlm.nih.gov/Blast.cgi). Seven putative *A. fumigatus* open reading frames predicted to encode a polysaccharide/chitin de-N-acetylase domain (Pfam 01522) were identified (http://pfam.xfam.org/) belonging to CE-4 family of the CaZy database (http://www.cazy.org:) *CDA1*, AFUA_1G15280; *CDA2*, AFUA_6G05030; *CDA3*, AFUA_3G07210; *CDA4*, AFUA_5G11410; *CDA5*, AFUA_4G09940, *CDA6*, AFUA_6G10430, and *CDA7*, AFUA_5G09130. The characteristics of these putative chitin deacetylases are given in Table S2. Conserved domains, including transmembrane domains and peptide signals, were characterized with SMART for each putative protein (http://smart.embl-heidelberg.de/). Cda3, Cda4, and Cda6 have a predicted N-terminal peptide signal, suggesting that these proteins are secreted while Cda1, Cda2, and Cda7 are predicted to be intracellular proteins. Cda6 contains a Carbohydrate Binding Domain (CBM18) and is likely GPI anchored (mendel.imp.ac.at/gpi/fungi). There was 4–20% of identity between all the putative Cda proteins except Cda2 and Cda7 which are very closely related (almost 80% of identity; Table S3). Amino acid conserved between all the Cdap are presented in Figure 3. A phylogenic tree containing all known and putative Cdas was created including the putative Cdap of *S. cerevisiae, S. pombe, C. neoformans*, and *M. oryzae* (Figure S2). Three distinct groups are observed: the first group including *A. fumigatus* Cda1, Cda2, and Cda7 with *S. pombe* Cda1p and *M. oryzae* Cbl5p, the second one including *A. fumigatus* Cda4, Cda5, and Cda6 related to all the putative Cdap of the yeast *S. cerevisiae, C. neoformans* and the other homologs of *M. oryzae*, and finally a third group including only *A. fumigatus* Cda3.

### Gene Level Expression of the CDA Family During Growth

**Figure 4:**
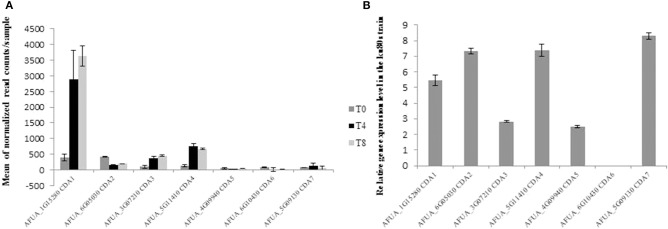
*CDA* gene expression level in *A. fumigatus* **(A)** Gene expression profile of the KU80 *CDA* family genes in dormant (T 0 h), swollen (T 4 h), and germinated conidia (T 8 h) in Glucose 3%/YE 1% medium. The data are extracted from RNAseq analysis data (Mouyna et al., 2016). The errors bars represent standard deviation from the mean values of three different experiments. The data have been normalized/size of the gene **(B)** Relative Gene expression level of the *CDA* genes in the mycelium during conidiation of the parental strain (WT). The expression ratios were relative to *TEF1* gene. Values are taken from the RNA-seq data analysis of Valsecchi et al. (2017). For this RNA-sequencing experiment, the fungus was grown for seven days on malt agar at 37°C at room temperature. The errors bars represent standard deviation from the mean values of three different experiments.

We used the expression data from a RNA sequencing analysis performed to quantify the expression of glycosyl-hydrolases in dormant, swollen, and germinated conidia (Mouyna et al., 2016; Figure 4A). In addition, since the level of glucosamine was the highest in the conidia, we undertook a specific analysis of the expression of the CDAs during the conidiation (Figure 4B). The gene *CDA1* was highly expressed especially in swollen and germinated conidia. Genes *CDA2, CDA3*, and *CDA4* were expressed in both stages, while *CDA7, CDA5*, and *CDA6* were only weakly expressed (Figure 4A). Genes *CDA1, CDA2, CDA7*, and *CDA4* were strongly expressed during conidiation (Figure 4B). The combined transcriptional profiling data suggested that *CDA1* may be involved in chitin deacetylation during germination and vegetative development and *CDA1, CDA2, CDA7*, and *CDA4* during conidiogenesis.

### Phenotypic Analysis of Δ*cda* Mutants

To determine the role of candidate *CDA* genes in chitosan synthesis, single deletion mutants for each *CDA* gene, and a septuple mutant lacking all seven *CDA* genes (Δ*cda1-7*) were constructed. Quantification of chitosan by free amine determination revealed no differences in the percentage of chitin deacetylation in the conidia of the Δ*cda1-7* mutant strain and the parental strain, suggesting that these seven candidate *CDA* genes do not play a role in the deacetylation of chitin in *A. fumigatus* conidia (Figure 2).

The effects of the deletion of putative *CDA* genes on *A. fumigatus* germination, growth and resistance to cell wall stress were evaluated. No differences in germination, growth in liquid or solid medium or biofilm growth were observed between wild-type *A. fumigatus* and the septuple Δ*cda1-7* mutant (Figure S3; data not shown). No difference in susceptibility to the ß1,3 glucan synthase inhibitor caspofungin was observed, with both strains exhibiting an MIC of 0.3 μg/mL. Similarly, agar dilution assays demonstrated that both strains displayed similar growth on media containing CR or CFW (data not shown). Moreover, no microscopic differences in hyphal morphology or branching pattern were observed between these strains in presence of any of these cell wall stressors (data not shown). These results confirmed that the chitosan does not play a role in the morphogenesis of the mycelial cell wall.

**Figure 5:**
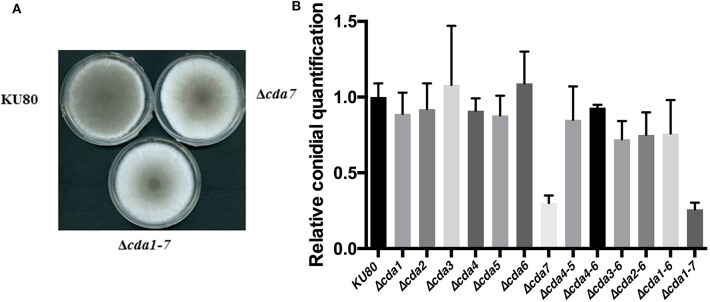
Vegetative growth of *A. fumigatus* wild-type strain and Δ*cda* mutants on GlcNAc as sole carbon source. **(A)** Colonies of the KU80, Δ*cda*7, and Δ*cda1-7* on GlcNAc-MM; **(B)** Conidia quantification on GlcNAc-MM. Data are normalized to parental strain KU80.

The effect of *CDA* deletions on conidiation was tested by examining the ability of the parental and Δ*cda1-7* mutant strains to conidiate on different solid media. No difference in conidiation of KU80 and Δ*cda1-7* was observed on regular glucose media (data not shown). However, when grown on GlcNAc as the sole carbon source (GlcNAc-MM), the Δ*cda1-7* mutant exhibited reduced conidiation, with 74 (±4.3)% fewer conidia produced as compared with the wild-type KU80 strain (*p* < 0.001) (Figures 5A,B). However, in presence of glucosamine conidiation was similar to the parental and other mutant strains (Figure S4). The conidiation defect of the Δ*cda1-7* mutant was uniquely dependent on the deletion of *CDA7*, since the single Δ*cda7* mutant exhibited a similar reduction in conidiation (70 ± 5.0%) as was observed in the septuple mutant (Figure 5B). In accordance with the observed conidiation defect of the Δ*cda7* and Δ*cda1-7* mutants, expression of *BrlA, WetA, and AbaA* were significantly down regulated in these strains (Figure S5). These results suggest that Cda7 may not function as a chitin deacetylase, but rather plays a role in GlcNAc-metabolism and/or sensing.

Since studies in plant pathogens suggest that the importance of the *CDAs* may be different *in vivo* and *in vitro*, we evaluated the role of putative *CDAs* in the pathogenesis of *Aspergillus* infection in a neutropenic mouse model of pulmonary invasive aspergillosis and in an *Aspergillus* corneal infection model in immunocompetent mice. No difference in the survival rate (Figure S6A) or pulmonary fungal burden of immunosuppressed mice infected by wild-type strain or the Δ*cda1-7* mutant strain were observed (Figure S6B). Similarly, there was no difference in fungal burden between these strains in infected corneas (Figure S7). Collectively these data showed that these candidate *CDA* genes are dispensable for the virulence of *A. fumigatus*.

## Discussion

In the plant pathogenic fungi *M. oryzae, P. graminis*, and *C. graminicola*, chitosan was located in the cell wall of the invasive and adhesive germ tube (El Gueddari et al., 2002; Geoghegan and Gurr, 2016). This observation initially suggested the possibility that chitosan in conidia and appressorium plays a role in host-adherence. In contrast, in *A. fumigatus* the amount of deacetylated chitin is minimal in the hyphal cell wall. This finding is in agreement with the lack of any role of the putative Cda proteins in vegetative growth or infection. These findings suggest that other positively charged-osamine polymers such as the ones present in GAG, mediate adhesion of *A. fumigatus* mycelium to host tissues (Lee et al., 2016).

In the conidia of *A. fumigatus*, we found that deacetylation of cell wall chitin was not due to the activity of the Cdas. Deacetylation of chitin in *A. fumigatus* conidial cell wall could be mediated by other *A. fumigatus* CE-4 family deacetylases such as Agd3, already known to deacetylate GalNac residues within GAG (Lee et al., 2016). CE-4 family included many other deacetylases with low specificity, which are active toward a variety of substrates. *A. nidulans* chitin deacetylase has been produced as a recombinant protein. It was able to fully deacetylate a partially acetylated chitosan and active only on chitin which is not-crystallized. As with other Cda, this protein exhibited poor substrate specificity as it was able to act on acetylated- xylan and glucuroxylan (Liu et al., 2017). The bacterial deacetylases, PelA in *Pseudomonas aeruginosa*, IcaB in *Staphylococcus epidermidis*, PgaB in *Escherichia coli* and PssB in *Listeria monocytogenes*, all homologous to the *A. fumigatus* Agd3, are also poorly specific since they are able to deacetylate different polymers of GlcNAC or N-acetylmannosamine residues (Lee et al., 2016; Ostapska et al., 2018).

Is deacetylated chitin necessary during the vegetative life of *A. fumigatus*? Probably not, since the deletion of *CDAs* did not alter the growth, germination, resistance to antifungal cell wall compounds, nor pathogenicity of *A. fumigatus*. However, the mutant deficient for *CDA7* showed impaired conidiation on MM containing GlcNAc as sole carbon source, which was confirmed in the septuple mutant. Cda7 is not a functional deacetylase since the same amount of deacetylated chitin was found in the sextuple mutant Δ*cda1-*7 but this esterase could be involved in GlcNAc catabolism necessary to produce primary carbon source for the fungal cell allowing normal conidial development. The function of AfCda7 is different from *A. nidulans's* Cda which deacetylated *in vitro* a minimum of two GlcNAc monomers (Liu et al., 2017). Another hypothesis is that Cda7 could be involved in glucosamine sensing. GlcNAc has been shown to activate intracellular signalization pathways through different mechanisms in microbial pathogens regulating different functions including virulence function in some species (Naseem and Konopka, 2015). For instance, it promotes hyphal growth in *Candida albicans*. GlcNAc catabolism increases medium pH by releasing ammonia, while glucose catabolism decreases the pH. A synergy between basic extracellular medium and GlcNAc recognition by GlcNAc sensors promotes hyphae formation in the yeast independently from hyphal-specific genes (Naseem and Konopka, 2015). *C. albicans* Gig1, another member of the CE-4 family is specifically induced by GlcNAc and not by other sugars (Gunasekera et al., 2010). Gig1 is not essential for GlcNAc metabolism or induction of hyphae but the Δ*gig1* mutant displayed increased resistance to nikkomycin Y, which inhibits chitin synthase from converting UDP-GlcNAc into cell wall chitin. Two orthologs with 20–26% of homology have been identified in the *A. fumigatus* genome. Yet, no protein homology was observed between Cda7 and the GlcNAc sensors such as Ngs1, the GlcNAc deacetylase Nag2 which is part of the NAG regulon governing the GlcNAc signalization pathway in *C. albicans*, nor Gig1 (Su et al., 2016; Naseem et al., 2017). The production of recombinant Cda proteins of *A. fumigatus*, as well as the analysis of the expression of the different *CDA*'s in presence of GlcNAc or GlcN will be required to elucidate the role of *CDAs* in *A. fumigatus*.

**Figure 6:**
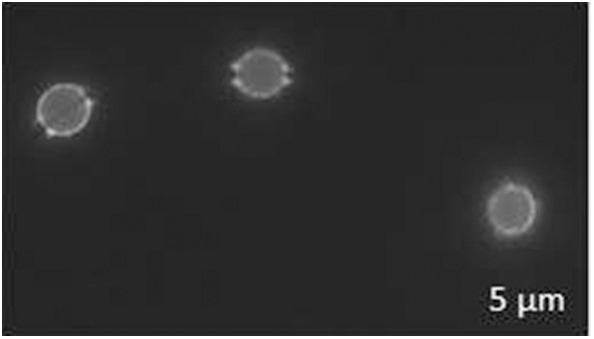
Ascospores of *A. fumigatus* labeled by Eosin Y.

*CDA*'s could also play a role in the formation of ascospores in *A. fumigatus*. Ascospores are the fungal structures with the highest proportion of chitosan (Zhang et al., 2017). In *S. cerevisiae, CDA*'s are specifically expressed during ascospore formation and chitosan appears to have a structural role (Pammer et al., 1992; Christodoulidou et al., 1996). The sexual form of *A. fumigatus* was recently discovered (O'Gorman et al., 2009). *A. fumigatus* ascospores have not been described in nature and require very specific conditions to be produced *in vitro* as they have been obtained only in oatmeal agar medium incubated in the dark at 30°C for several months before sexual reproduction was completed. *A. fumigatus* ascospores were eosin Y positive, showing that they contained substantial amount of chitosan (Figure 6). *CDA*'s could be responsible for the deacetylation of chitin in *A. fumigatus* ascospores and defect in chitosan may inhibit ascospore formation like in *S. cerevisiae* (Pammer et al., 1992).

In conclusion, despite the fact that chitin deacetylases are present throughout the Fungi kingdom and exhibit similar catalytic sites in their protein sequences, they appeared to mediate very different biological processes depending on the organism. In *A. fumigatus*, our study suggests that chitin in conidia was not deacetylated by Cda proteins. The enzyme responsible for deacetylation of chitin within the conidial cell wall is still unknown but could be due to other CE4 deacetylases. In *A. fumigatus* the deletion of *CDA* genes did not play any role in morphogenesis, cell wall integrity, adherence and virulence. While *A. fumigatus* is able to produce ascospores with a chitosan-rich cell wall, the involvement of *CDA*s for the formation of chitosan in these structures remains to be demonstrated.

## Data Availability Statement

The raw data supporting the conclusions of this article will be made available by the authors, without undue reservation, to any qualified researcher.

## Ethics Statement

All procedures involving mice were approved by the Animal Care Committees of the McGill University Health Center and followed the National Institutes of Health guidelines for animal housing and care.

## Author Contributions

IM, SD, AB, EP, DS, and J-PL conceived and designed the experiments, analyzed the data, and wrote the paper. IM, SD, AB, FG, BS, ML, CZ, YS, and SJ performed the experiments.

### Conflict of Interest

The authors declare that the research was conducted in the absence of any commercial or financial relationships that could be construed as a potential conflict of interest.

## Supplementary Material

Figure S1 Targeted replacement strategies used for *A. fumigatus CDA* genes.

Figure S2 Phylogeny of the proteins of Af, *A. fumigatus*; Mo, *M. oryzae*; Sc, *S. cerevisiae*; Cn, *C. neoformans*; Sp, *S. pombe* and *Mucor* rouxii. Sequence alignment and phylogenetic reconstructions have been done using clustalW (https://www.genome.jp/tools-bin/clustalw) The tree was constructed using FastTree v2.1.8 with default parameters (Price et al., 2009).

Figure S3 Growth on agar Malt medium of the parental strain KU80, the single deletion Δ*cda* mutants as well as the Δ*cda1-7* deletion mutant.

Figure S4 Growth on agar glucosamine-MM medium of the parental strain KU80, the single deletion Δ*cda7* mutants as well as the Δ*cda1-7* deletion mutant.

Figure S5 Transcription factor gene expression level by QPCR on GlcNAc-MM medium. Gene expression data expressed as 2^−ΔΔ*Ct*^ are the mean of at least three replicates ± standard error and were calculated as the ratio of the target gene compared with the Elongation factor 1 (EF1α) gene used as a reference.

Figure S6 Virulence of Δ*cda* deficient strain in a mice model of invasive aspergillosis. A total of 16 animals were infected by each strain. **(A)** Survival of immunosuppressed mice infected intranasally with *A. fumigatus* parental strain KU80 and Δ*cda1-7* mutant and of uninfected strain **(B)** Quantification of fungal burden in lung homogenates of mice after 4 days of infection using pulmonary galactomannan content as a surrogate measure. Error bars indicate standard errors.

Figure S7 Role of the Cdas in *A. fumigatus* survival in corneal infection. CFU of infected corneas for the parental strain KU80 and the Δ*cda 1-7* mutant. Each data point represents a single infected cornea. Significance was determined by student's *T*-test to compare two groups. *P*-values: ^*^ < 0.05, ^***^ < 0.0001. Experiments were repeated three times with similar results.

Table S1 Primers used in this study.

Table S2 Characteristics of *A. fumigatus CDA* family.

Table S3 Percent of identity between the *A. fumigatus CDA* family members, *CDA* from *C. lindemuthianum* (Cl CDA-col) and *CDA1* from *S. cerevisiae* (Sc), *S. pombe* (Sp), *C. neoformans* (Cn), and *M. grisea* (Mg). The% of identities between these proteins have been calculated after sequence alignment using clustalW (https://www.genome.jp/tools-bin/clustalw) and GeneDoc 2.7 software.
