# Supplementary material for: What Are the Functions of Chitin Deacetylases in Aspergillus fumigatus?
Source: Front Cell Infect Microbiol. 2020 Feb 6;10:28. doi: 10.3389/fcimb.2020.00028 (PMC7016196; doi:10.3389/fcimb.2020.00028)
Supplement: Figure S1 — Targeted replacement strategies used for A. fumigatus CDA genes. [file Presentation_1.pptx]

## Slide 1
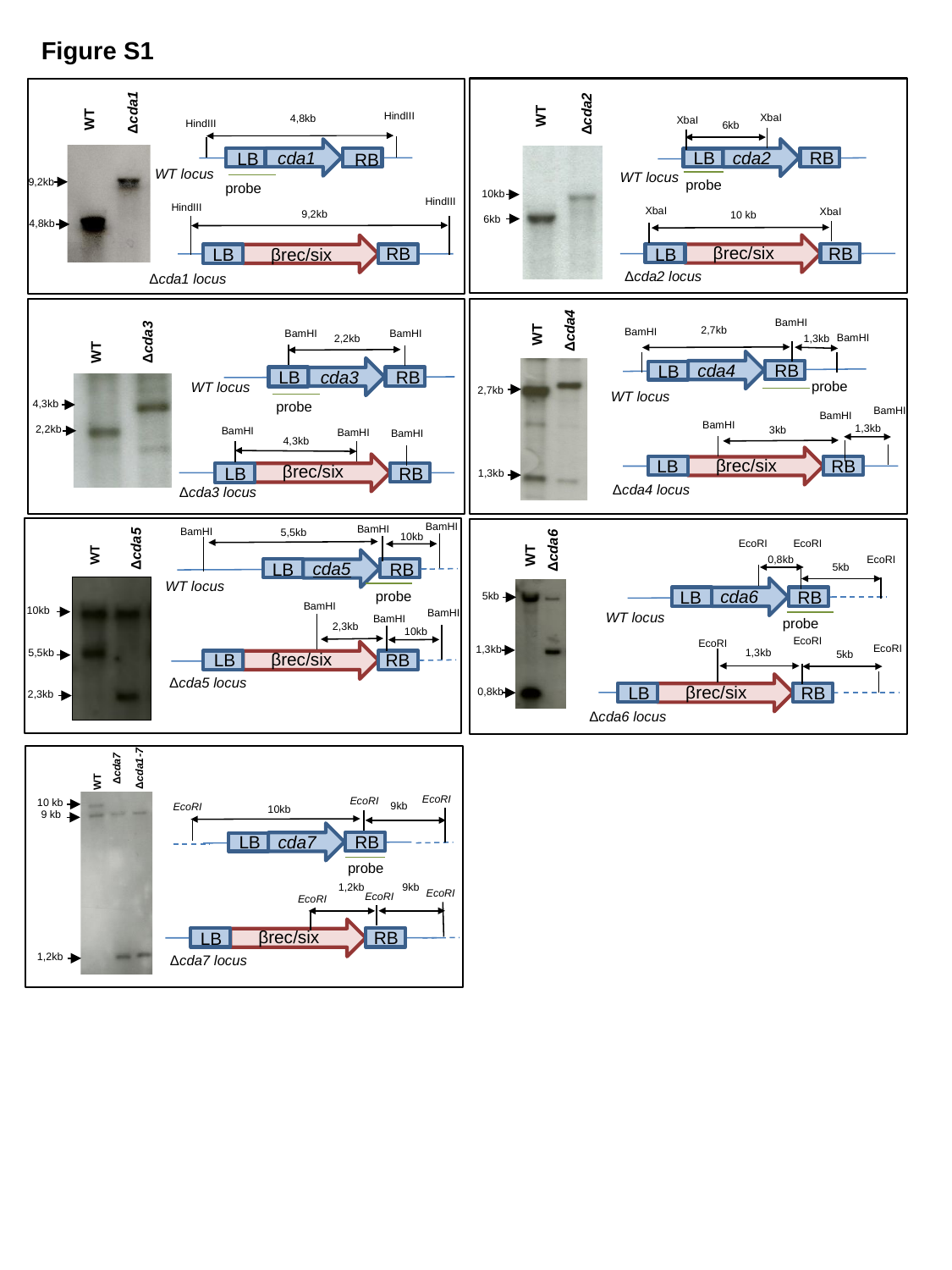

Figure S1
Δcda1
Δcda2
WT
HindIII
XbaI
WT
4,8kb
XbaI
HindIII
6kb
LB
cda2
cda1
RB
LB
RB
WT locus
WT locus
9,2kb
probe
probe
10kb
HindIII
HindIII
XbaI
XbaI
9,2kb
10 kb
6kb
4,8kb
RB
LB
βrec/six
RB
LB
βrec/six
Δcda2 locus
Δcda1 locus
BamHI
Δcda4
2,7kb
BamHI
WT
BamHI
BamHI
BamHI
1,3kb
2,2kb
Δcda3
WT
cda4
RB
LB
RB
cda3
LB
probe
WT locus
2,7kb
WT locus
4,3kb
probe
BamHI
BamHI
BamHI
1,3kb
2,2kb
3kb
BamHI
BamHI
BamHI
4,3kb
LB
RB
βrec/six
βrec/six
RB
LB
1,3kb
Δcda4 locus
Δcda3 locus
BamHI
BamHI
BamHI
5,5kb
10kb
EcoRI
EcoRI
WT
Δcda5
Δcda6
WT
EcoRI
0,8kb
cda5
LB
RB
5kb
WT locus
cda6
LB
RB
probe
5kb
BamHI
10kb
BamHI
WT locus
BamHI
probe
2,3kb
10kb
EcoRI
EcoRI
EcoRI
1,3kb
1,3kb
5,5kb
5kb
βrec/six
LB
RB
Δcda5 locus
βrec/six
LB
RB
0,8kb
2,3kb
Δcda6 locus
Δcda7
Δcda1-7
WT
EcoRI
EcoRI
10 kb
9kb
EcoRI
10kb
9 kb
LB
cda7
RB
probe
1,2kb
9kb
EcoRI
EcoRI
EcoRI
RB
LB
βrec/six
1,2kb
Δcda7 locus

## Slide 2
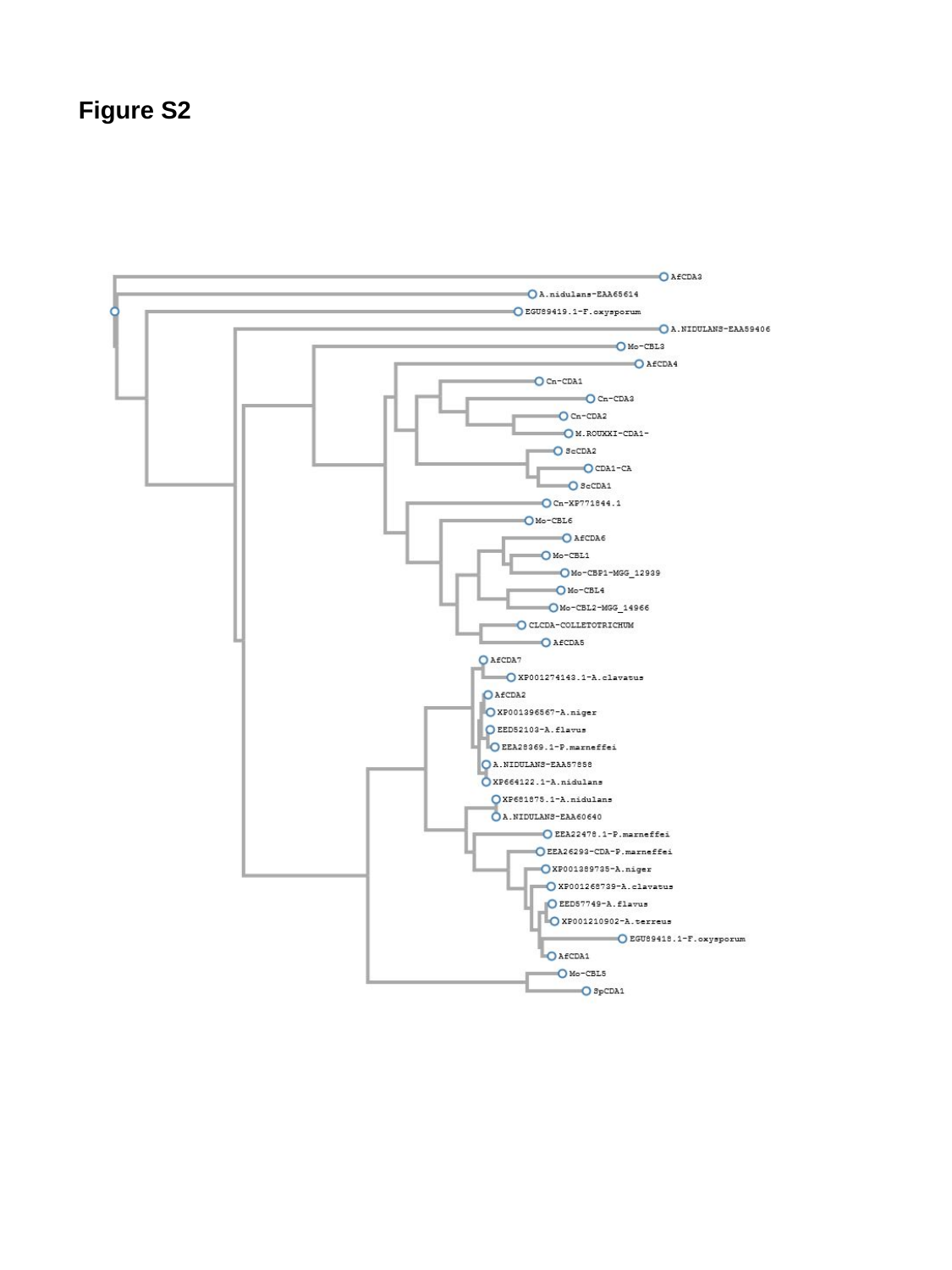

Figure S2

## Slide 3
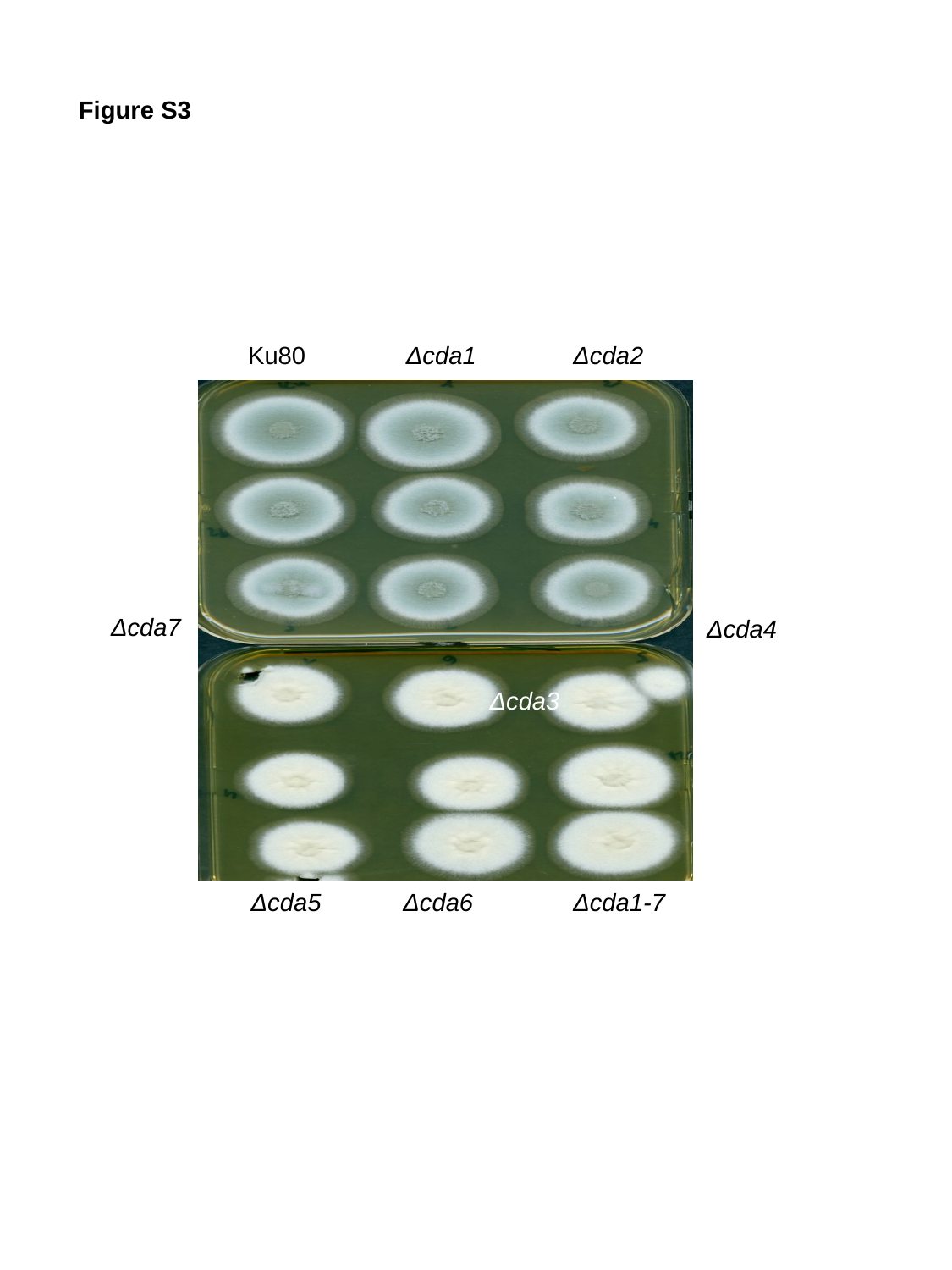

Figure S3
Ku80
Δcda1
Δcda2
Δcda7
Δcda4
Δcda3
Δcda5
Δcda6
Δcda1-7

## Slide 4
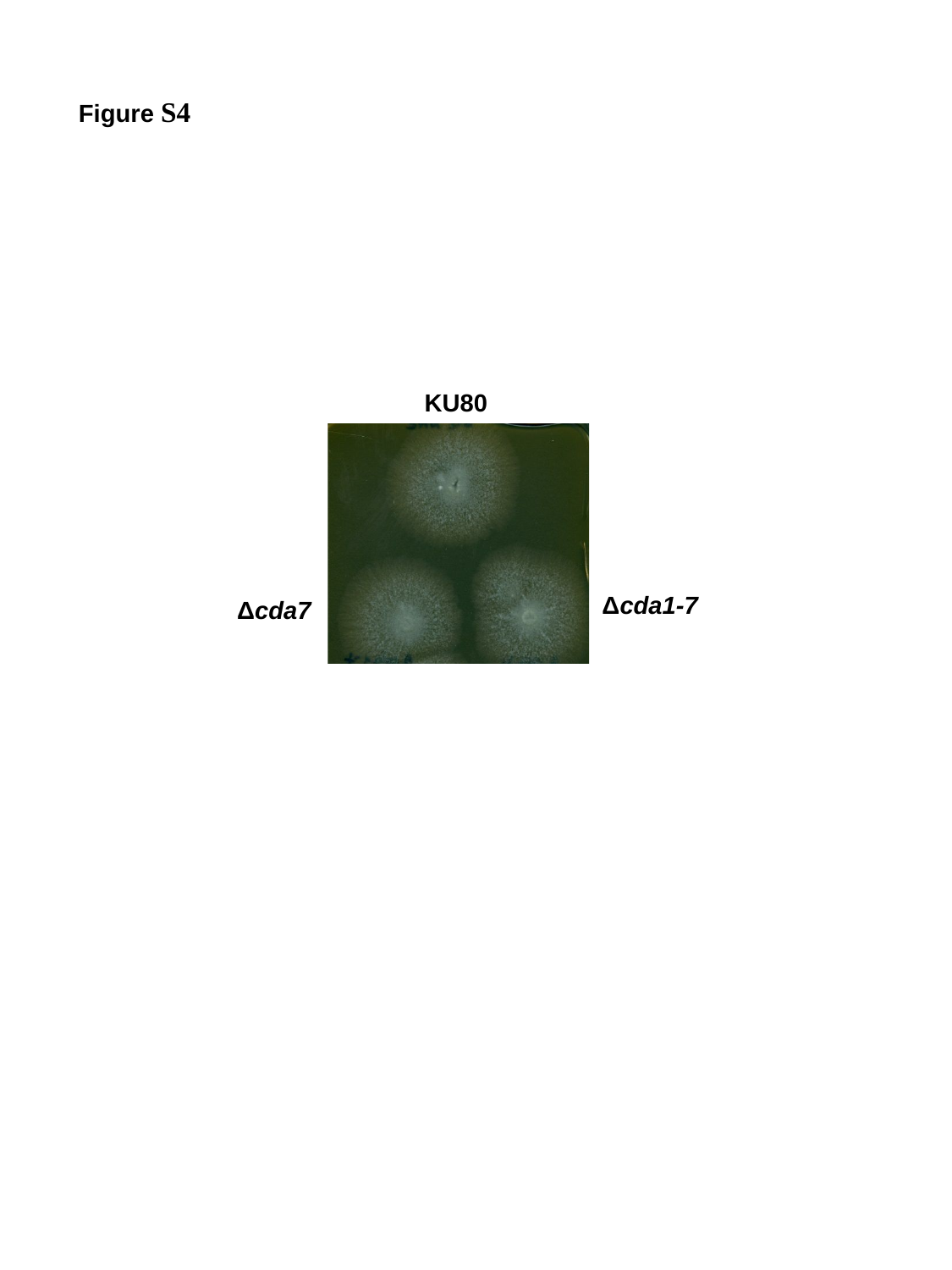

Figure S4
KU80
Δcda1-7
Δcda7

## Slide 5
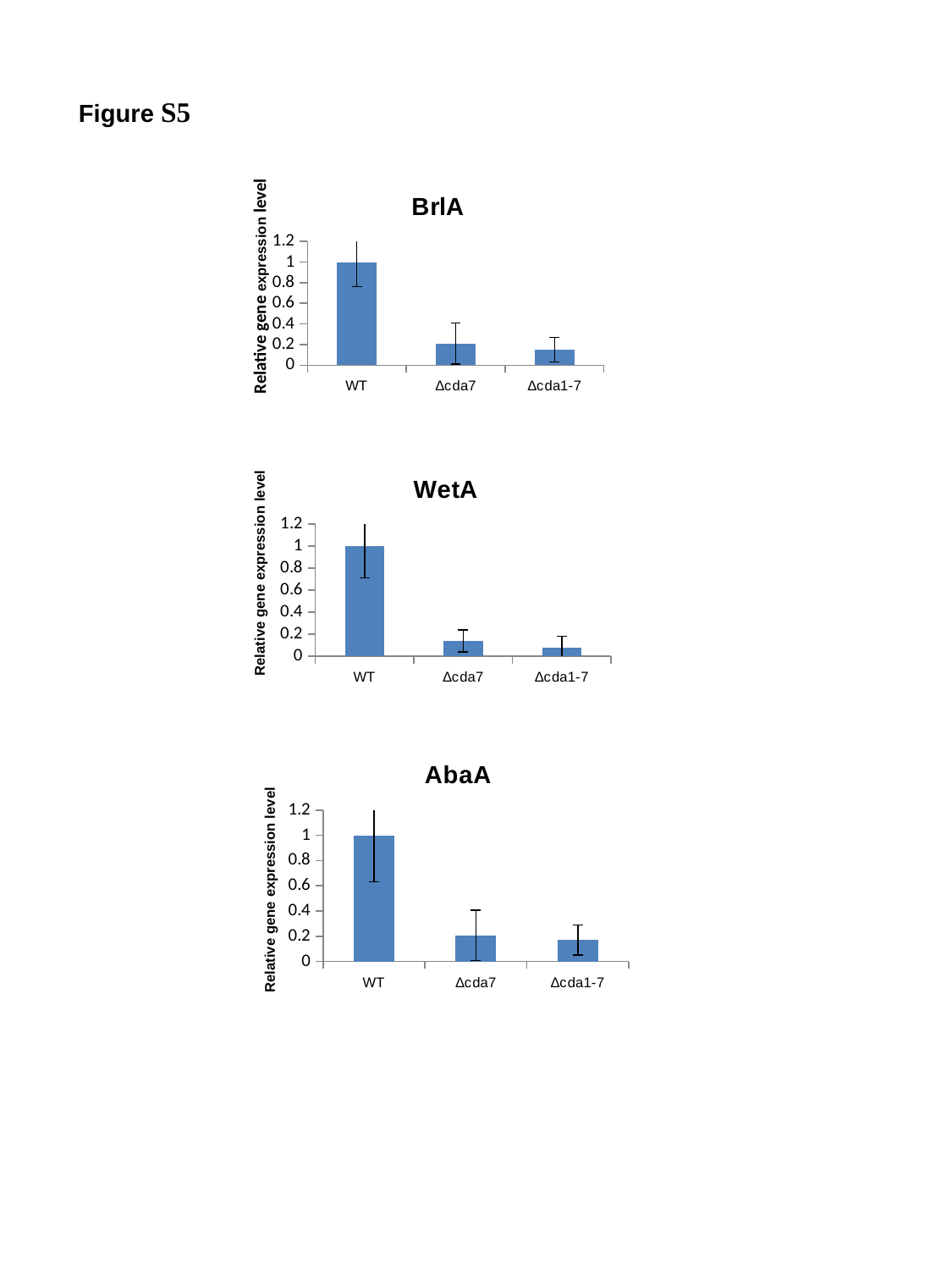

Figure S5
### Chart:
| Category | BrlA |
|---|---|
| WT | 1.0 |
| Δcda7 | 0.21 |
| Δcda1-7 | 0.15 |Relative gene expression level
### Chart:
| Category | WetA |
|---|---|
| WT | 1.0 |
| Δcda7 | 0.14 |
| Δcda1-7 | 0.08 |Relative gene expression level
### Chart:
| Category | AbaA |
|---|---|
| WT | 1.0 |
| Δcda7 | 0.208 |
| Δcda1-7 | 0.17 |Relative gene expression level

## Slide 6
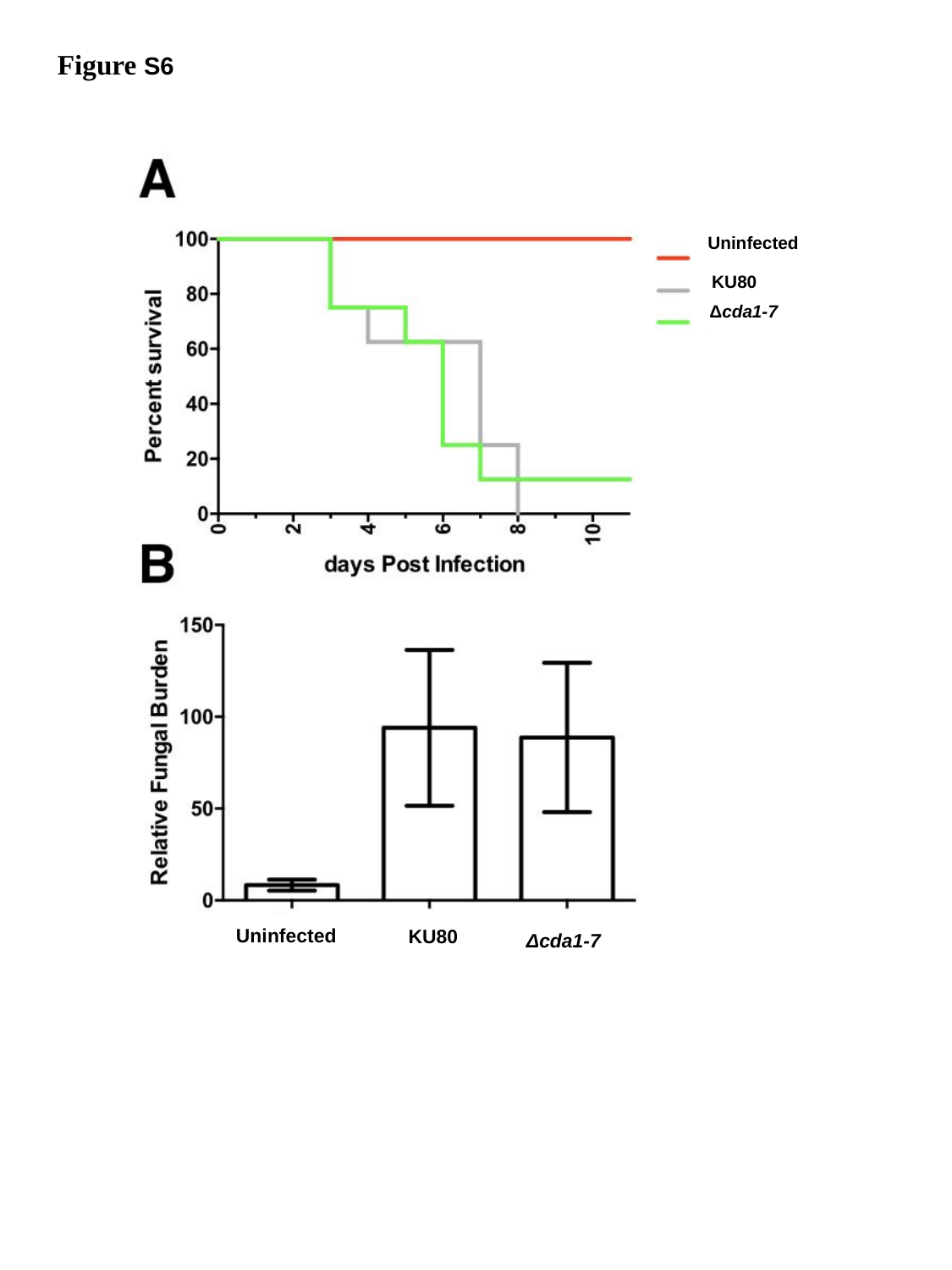

Figure S6
Uninfected
KU80
Δcda1-7
Uninfected
KU80
Δcda1-7

## Slide 7
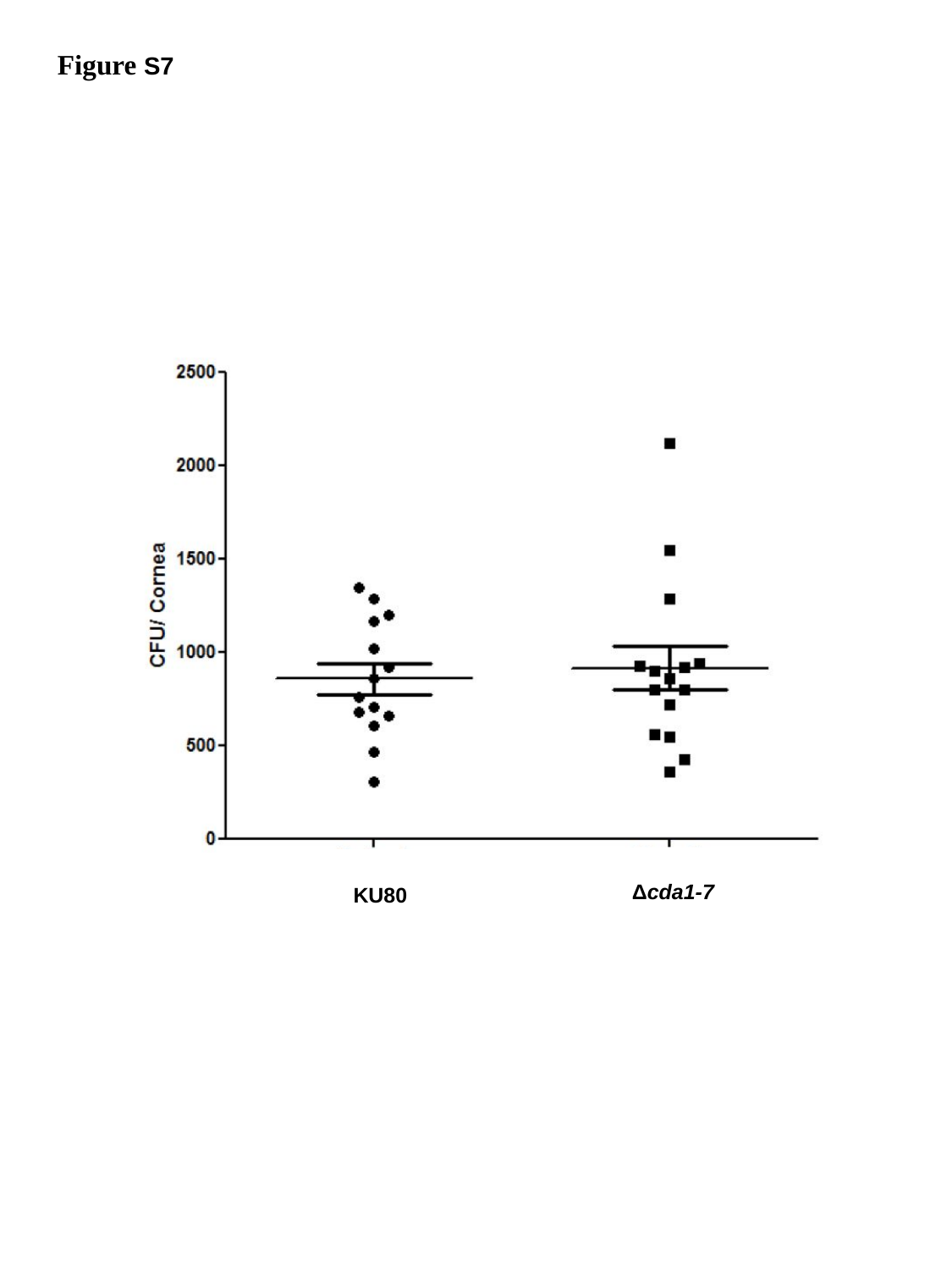

Figure S7
Δcda1-7
KU80
