# Supplementary material for: What Are the Functions of Chitin Deacetylases in Aspergillus fumigatus?
Source: Front Cell Infect Microbiol. 2020 Feb 6;10:28. doi: 10.3389/fcimb.2020.00028 (PMC7016196; doi:10.3389/fcimb.2020.00028)
Supplement: Table S1 — Primers used in this study. [file Data_Sheet_1.docx]

**Table S1:** Primers used in this study

| **NAME** | **SEQUENCE 5’🡪 3’** | USE |
| --- | --- | --- |
| CDA1F1 | AATTCGAGCTCGGTAC**CCCGGG**TTGCACGGCTAAGTGATGAG | Construction of the deletion *CDA1* cassette  with SmaI |
| CDA1R1 | GGACCTGAGTGATGCGAGTCTCTCCAGGCTGGTTG |  |
| CDA1F2 | TGGTCCATCTAGTGCACAACATCCTCGCCGACTAC |  |
| CDA1R2 | GCCAAGCTTGCATGCC**CCCGGG**AAAAAGCGTTTTGCTTGGAA |  |
| CDA2F1 | AATTCGAGCTCGGTAC**TGCGCA**GCATCATCCGCTCGGTACTA | Construction of the deletion *CDA2* cassette  with FspI |
| CDA2R1 | GGACCTGAGTGATGCTCGTTCGAGCTATCCTCACC |  |
| CDA2F2 | TGGTCCATCTAGTGCAGCAAAAGGACGTTCTGGAC |  |
| CDA2R2 | GCCAAGCTTGCATGCC**TGCGCA**AACGGACGAGAAGTCTGAGC |  |
| CDA3F1 | AATTCGAGCTCGGTAC**TGCGCA**GGCTGTCTTTTTGGACCTCA | Construction of the deletion *CDA3* cassette  with *FspI* |
| CDA3R1 | GGACCTGAGTGATGCTGTTCTGGTTGAGCGAGATG |  |
| CDA3F2 | TGGTCCATCTAGTGCAAACAGGCTAGCCAACGGTA |  |
| CDA3R2 | GCCAAGCTTGCATGCC**TGCGCA**TTGATTCTCCGTTCCATTCC |  |
| CDA4F1 | AATTCGAGCTCGGTAC**TGCGCA**CAGCATCATGGGATTCCTCT | Construction of the deletion *CDA4* cassette  with *FspI* |
| CDA4R1 | GGACCTGAGTGATGCGCCTTTAGCGTTGCTGCTAT |  |
| CDA4F2 | TGGTCCATCTAGTGCTGAGAGGATGAGGAGGTTGG |  |
| CDA4R2 | GCCAAGCTTGCATGCC**TGCGCA**GAGTTCACGGTGTGTGAGGA |  |
| CDA5F1 | AATTCGAGCTCGGTAC**TGCGCA**TAGCTGGGATTCGGAAAATG | Construction of the deletion *CDA5* cassette  with *FspI* |
| CDA5R1 | GGACCTGAGTGATGCAAGGTGGCCTCATGTACGTC |  |
| CDA5F2 | TGGTCCATCTAGTGCAGTTCCGTGCAGAGTTGGAT |  |
| CDA5R2 | GCCAAGCTTGCATGCC**TGCGCA**GGTCCAATATTCAGTGGAGCA |  |
| CDA6F1 | AATTCGAGCTCGGTAC**TGCGCA**GTGCCGTCTGTTCTTTCCTC | Construction of the deletion *CDA6* cassette  with *FspI* |
| CDA6R1 | GGACCTGAGTGATGCAGACTTGAATGGCGCTAGGA |  |
| CDA6F2 | TGGTCCATCTAGTGCGACGCTCCTTCGACTGTTTC |  |
| CDA6R2 | GCCAAGCTTGCATGCC**TGCGCA**CGCAAGCTCCTATCAAGGAC |  |
| CDA7F1 | AATTCGAGCTCGGTAC**TGCGCA**cgcgatgtcttggaaagatt | Construction of the deletion *CDA7* cassette  with FspI |
| CDA7R1 | GGACCTGAGTGATGCCATGTCCTTCGGGTTCTCAT |  |
| CDA7F2 | TGGTCCATCTAGTGCCACGAAGACTGCCAAGCATA |  |
| CDA7R2 | GCCAAGCTTGCATGCCTGCGCAGAAGACGGGGAAGGCTAAAC |  |
| BRLAqa | CGGATTTCTCCAACTCTATC | Expression of *BRLA* |
| BRLAqb | ATTCATCCATGTACTCACCA | Expression of *BRLA* |
| ABAAqa | AGCAAAGCCGAAGATGGACTACC | Expression of *ABAA* |
| ABAAqb | GCTGGAGACATTGAGACTGGATGG | Expression of *ABAA* |
| WETAqa | ATGCGACTCCTCTCCCGTTCAAC | Expression of *WETA* |
| WETAqb | ACAACTTCCTGCCGTGGTAGC | Expression of *WETA* |
| TEF1qa | CCATGTGTGTCGAGTCCTTC | Expression of *TEF1* |
| TEF1qb | GAACGTACAGCAACAGTCTGG | Expression of *TEF1* |

**Table S2**: Characteristics of the *A. fumigatus CDA* family

| Genes | Family | Size (aa) | SP |
| --- | --- | --- | --- |
| *CDA1* AFUA_1G15280 | CE4 | 584 | No |
| *CDA2* AFUA_6G05030 | CE4 | 305 | No |
| *CDA3* AFUA_3G07210 | CE4 | 452 | Yes |
| *CDA4* AFUA_5G11410 | CE4 | 260 | Yes |
| *CDA5* AFUA_4G09940 | CE4 | 254 | Yes |
| *CDA6* AFUA_6G10430* | CE4 | 487 | Yes and CBM18 |
| *CDA7* AFUA_5G09130 | CE4 | 308 | No |

Aa: amino acid; SP: Signal Peptide; *GPI anchored protein

**Table S3**: Percent of identity between the *A. fumigatus CDA* family members, *CDA* from *C.* *lindemuthianum* (*Cl CDA-col: AY633657*)

and *CDA1* from *S. cerevisiae*, (Sc) *S. pombe*, (Sp) *C. neoformans* (Cn) and *M. grisea* (Mg). The % of identities between these proteins have been calculated after sequence alignment using clustalw and GeneDoc 2.7 software.

|  | ***CDA2*** | ***CDA7*** | ***CDA3*** | ***CDA4*** | ***CDA5*** | ***CDA6*** | ***ClCDA-col*** | ***Sc CDA1*** | ***Sp CDA1*** | ***Cn CDA1*** | ***Mg CDA1*** |
| --- | --- | --- | --- | --- | --- | --- | --- | --- | --- | --- | --- |
| ***CDA1*** | **19** | 19 | 4 | 3 | 5 | 6 | 7 | 5 | 5 | 6 | 7 |
| ***CDA2*** |  | **80** | 3 | 5 | 6 | 7 | 8 | 6 | 6 | 6 | 5 |
| ***CDA7*** |  |  | 4 | 5 | 6 | 7 | 8 | 6 | 6 | 6 | 6 |
| ***CDA3*** |  |  |  | 6 | 9 | 8 | 9 | 10 | 5 | 11 | 8 |
| ***CDA4*** |  |  |  |  | 19 | 10 | 17 | 10 | 6 | 7 | 7 |
| ***CDA5*** |  |  |  |  |  | 20 | 40 | 17 | 9 | 11 | 18 |
| ***CDA6*** |  |  |  |  |  |  | 20 | 10 | 7 | 12 | 36 |
| ***ClCDA*** |  |  |  |  |  |  |  | 16 | 7 | 12 | 18 |
| ***Sc CDA1*** |  |  |  |  |  |  |  |  | 6 | 11 | 10 |
| ***Sp CDA1*** |  |  |  |  |  |  |  |  |  | 5 | 5 |
| ***Cn CDA1*** |  |  |  |  |  |  |  |  |  |  | 12 |
